# Aseptic Meningitis Linked to *Borrelia afzelii* Seroconversion in Northeastern Greece: An Emerging Infectious Disease Contested in the Region

**DOI:** 10.3390/tropicalmed9010025

**Published:** 2024-01-19

**Authors:** Dimitrios Kouroupis, Maria Terzaki, Nikoletta Moscha, Anastasia Sarvani, Elisavet Simoulidou, Sofia Chatzimichailidou, Evangelia Giza, Georgios Sapouridis, Emmanouil Angelakis, Konstantinos Petidis, Athina Pyrpasopoulou

**Affiliations:** 1 2nd Propedeutic Department of Internal Medicine, Hippokration Hospital, 54642 Thessaloniki, Greece; dimcour841@gmail.com (D.K.); mary.terz@hotmail.com (M.T.); nickol512@yahoo.com (N.M.); natasasar91@gmail.com (A.S.); elisavetsim@gmail.com (E.S.); sofia_hatzi75@hotmail.com (S.C.); petidisk@med.auth.gr (K.P.); 2 Neurology Department, Hippokration Hospital, 54642 Thessaloniki, Greece; evelynaris@gmail.com; 3 Department of Radiology, Hippokration Hospital, 54642 Thessaloniki, Greece; gsap79@hotmail.com; 4 Hellenic Pasteur Institute, 11521 Athens, Greece; e.angelakis@hotmail.com

**Keywords:** Lyme disease, *Borrelia afzelii*, aseptic meningitis, seroconversion

## Abstract

Borreliosis (Lyme disease) is a zoonosis, mediated to humans and small mammals through specific vectors (ticks), with increasing global incidence. It is associated with a variety of clinical manifestations and can, if not promptly recognized and left untreated, lead to significant disability. In Europe, the main *Borrelia* species causing disease in humans are *Borrelia burgdorferi s.s.*, *Borrelia afzelii*, *Borrelia garinii*, and *Borrelia spielmanii*. The *Ixodes ricinus* tick is their principal vector. Although Lyme disease is considered endemic in the Balkan region and Turkey, and all three main Lyme pathogens have been detected in ticks collected in these countries, autochthonous Lyme disease remains controversial in Greece. We report a case of aseptic meningitis associated with antibody seroconversion against *Borrelia afzelii* in a young female patient from the prefecture of Thasos without any relevant travel history. The patient presented with fever and severe headache, and the cerebrospinal fluid examination showed lymphocytic pleocytosis. Serum analysis was positive for specific IgG antibodies against *Borrelia afzelii*. In the absence of typical erythema migrans, serological evidence of infection is required for diagnosis. Although atypical in terms of clinical presentation, the seasonality and geographical location of potential disease transmission in the reported patient should raise awareness among clinicians for a still controversial and potentially underreported emerging infectious disease in Greece.

## 1. Introduction

Lyme disease is an infectious syndrome caused by various species of *Borrelia*, a genus of bacteria of the spirochete phylum. It is a zoonotic, vector-borne disease transmitted to humans from vertebrate reservoirs through tick bites [1]. Lyme disease occurs regularly in the temperate regions of the northern hemisphere. It is the most commonly reported tick-borne illness in North America, with an estimated incidence of 476,000 cases annually, and is considered endemic throughout most of central Europe and parts of Asia. The prevalence of tick-borne diseases in general, and Borreliosis in particular, is rising and spreading to previously unaffected areas due to several biotic and abiotic factors [2], climate change perhaps being the one with the most significant imprint. Seasonality has shifted weeks earlier, both in North America as well as in Europe, and this may well be associated with increased temperature and the moisture-associated acceleration of the tick life cycle [3]. People engaged in outdoor recreational activities in forested areas, such as campers, hikers, and hunters, are per definition mostly at risk of becoming infested by the arthropod vectors and contracting the bacterium. Thus, the growing tendency to create natural environments and promote recreational activities in nature in combination with favorable environmental and climatic factors increases the probability of unsuspected visitors becoming exposed to ticks vectoring infectious agents [4].

Besides the main actually clinically involved *Borrelia* species differentiation between North America and Europe (*Borrelia burgdorferi* vs. *Borrelia afzelii*, *Borrelia garinii*, and *Borrelia spielmanii*, respectively), presentation also varies. In Europe, Acrodermatitis chronica atrophicans and *Borrelia lymphocytoma* are encountered more often, and neuroborreliosis is more frequent, presenting mainly as cranial nerve palsy, particularly facial nerve palsy, lymphocytic meningitis, and painful radiculitis [5]. Neuroborreliosis indeed appears to rank as one of the most frequent bacterial central nervous system (CNS) infections in Europe [6].

The heterogeneity of clinical manifestations associated with the diverse *Borrelia* species worldwide leads to regional differences in terms of diagnosis. Diagnosis is currently based on serology using two tests (two-tiered protocol): an enzyme-linked immunosorbent assay (ELISA) and Western blot (WB) with, however, significant caveats, mainly the lack of sensitivity in the early stages of the disease and potential cross-reactivity issues [7]. We describe the interesting case of probable Lyme disease with atypical features in a geographical region of Northern Greece, in which the emergence of Borreliosis is anticipated, but not established to date, and discuss on one hand the significance of maintaining a high clinical suspicion in a changing planet and, on the other, the difficulties establishing such a diagnosis.

## 2. Detailed Case Description

In late July, a 21-year-old female patient from Thasos Island in Northeastern Greece was referred to the Emergency Department with a progressively worsening headache of 14 days duration and intermittent low-grade fever. On the day of presentation, the patient had attended the health center of her region of origin, where she was initially treated with IV paracetamol and diazepam. Upon referral, the patient had low-grade fever, complained of a severe intense headache with photophobia, vomited prior to presentation, and was slightly confused. The patient’s history was not positive for any chronic illnesses; she mentioned having occasional migraines with different clinical features compared to her presenting symptomatology.

**Figure 1:**
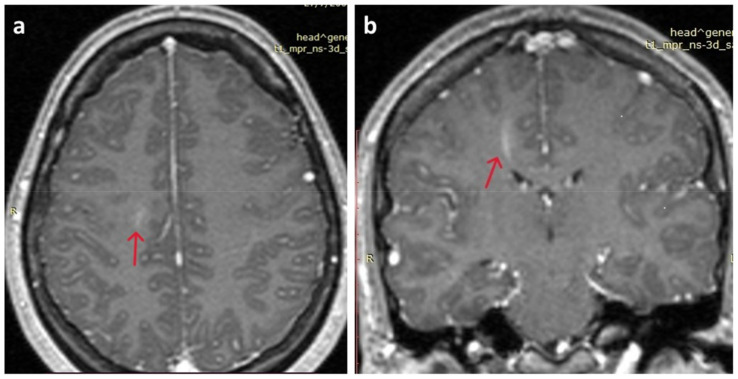
Brain MRI. (**a**) Mild leptomeningeal enhancement in both brain hemispheres (red arrow); (**b**) linear enhancing formation in the right centrum semiovale, characterized as a probable venous angioma S (red arrow).

At presentation, neurological assessment did not reveal any focal neurological signs, except for mild meningism with a positive Brudzinski’s sign. Laboratory tests were within the normal limits, as were inflammatory markers. The chest X-ray was unremarkable. A brain CT scan did not reveal any acute pathology. A lumbar puncture was performed, which revealed CSF lymphocytic pleocytosis (350 lymphocytes/μL), mildly elevated protein 69mg/dl (nv < 45 mg/dL), glucose 59mg/dL (within normal limits), and normal LDH, 19mg/dl. Treatment was initiated with IV acyclovir, ceftriaxone, and analgesics. Film array (multiplex PCR) of the CSF did not detect any of the common meningitis/encephalitis pathogens, and CSF did not grow any bacterial or fungal pathogens. Brain MRI was unremarkable, except for leptomeningeal enhancement in both brain and cerebellar hemispheres and a linear enhancing formation in the right centrum semiovale, characterized as a probable venous angioma, which was reviewed by the neurosurgeons and was not considered to be clinically significant (Figure 1). The electroencephalogram (EEG) was within normal limits. Ophthalmological examination (including fundoscopy) was also normal. Muscle relaxant medication (orphenadrine citrate) was added to her treatment, and the antimicrobial/antiviral treatments were discontinued.

Serology for *West Nile virus* and syphilis in peripheral blood and PCR for enteroviruses in the CSF were negative; HIV antigen and antibodies were undetectable. The patient remained in hospital under treatment and improved quickly. A series of autoantibodies were negative; low-positive titer of rheumatoid factor, anti-citrullinated peptides, and anti-Ro autoantibodies were detected initially, which were undetectable upon retesting (one month later). Prior to discharge, a serum sample against *Borrelia* serology was tested with Western blot (WB) at the national reference laboratory. The result was equivocal, with two IgG genus-specific antibodies testing positive (p21 and OspC) and one marginally positive IgG antibody against *Borrelia afzeli.* It was recommended to re-test the patient after a period of one month. The patient was discharged on oral doxycycline for two weeks. One month later, at re-evaluation, she was symptom-free. A new serology sample was taken and tested against *Borrelia* both with an enzyme-linked immunosorbent assay (ELISA) and Western blot (WB). The ELISA was positive for IgG antibodies against *Borrelia* (1.72 IU/mL, negative < 1.1) and marginally positive (grey zone) for IgM antibodies. Western blot was at re-evaluation was characterized as positive with four IgG genus-specific antibodies testing positive (p19, p20, p21, and OspC) and one positive IgG antibody against *Borrelia afzelii*. The remaining CSF sample collected during the initial admission was negative, as indicated by PCR, for *Borrelia*, and no intrathecal antibody production was detected. Repeat brain MRI showed improvement in leptomeningeal enhancement. The probability of neuroborreliosis could not be fully excluded due to CNS pleocytosis and the symptomatology. A follow-up treatment course of two weeks IV ceftriaxone was further administered. The patient was unwilling to undergo a new lumbar puncture. 

## 3. Discussion

Our patient presented as a typical case of meningoencephalitis, and in the process of further investigating the cerebrospinal fluid (CSF) lymphocytic pleocytosis associated with her symptomatology, positive, specific serology for *Borrelia afzeli* was detected in the absence of other potential pathogenic contributors. The possible diagnosis of Lyme neuroborreliosis was further pursued, but both PCR tests for *Borrelia* performed on CSF were negative, and intrathecal antibody production was not confirmed. Neurological involvement (Lyme neuroborreliosis) of both the central and peripheral nervous systems can occur in up to 15% of *Borrelia* patients with early disseminated disease [8]. Lyme neuroborreliosis occurs usually in early disseminated disease within a few weeks of infection. Presentation involves (in adults) mainly painful meningoradiculoneuritis (Garin–Bujadoux–Bannwarth syndrome) and unilateral or bilateral facial palsy separately or together. Rare presentations include meningitis, myelitis, encephalitis, cerebral vascu-litis, and stroke [9]. To reach a definite neuroborreliosis diagnosis, three out of the following criteria (two in the case of possible neuroborreliosis) need to be fulfilled: presence of neurological symptoms; inflammatory characteristics of the cerebrospinal fluid (CSF), e.g., lymphocytic pleocytosis; and *Borrelia*-specific antibodies produced intrathecally. Molecular testing (PCR) and CSF culture may be corroborative if symptoms duration is less than 6 weeks, when serology may still be negative or in patients with immunodeficiency [10], but they are otherwise not recommended because of their low sensitivity and undetermined specificity. Our patient did not fulfil all the necessary criteria to be indisputably characterized as a definite neuroborreliosis case. However, the presence of specific *Borrelia* antibodies and the course of their seroconversion within the context of her symptomatology render her at least a very probable case of Borreliosis in a region with unknown prevalence of the disease. The probability of the diagnosis is fortified by the absence of other potentially implicated pathogens or other pathological conditions. It was therefore treated as a Borreliosis case with associated aseptic meningitis in a region/country in which no officially confirmed cases have been recorded. 

Lyme borreliosis (LB) is the most common arthropod-borne disease in temperate regions of the northern hemisphere. Due to the biology of transmission, the relative risk of infection is higher in association with outdoor occupations (e.g., forestry work) and certain outdoor recreational activities (e.g., specific sports or mushroom collection) [11]. In Europe, *Borrelia afzelii, Borrelia garinii,* and *Borrelia spielmanii* genospecies of the spirochaete *Borrelia burgdorferi sensu lato* complex are the main causative pathogens of Lyme disease. The bridge vectors (vectors that feed on more than one host species) that transmit *Borrelia burgdorferi sensu lato complex* species to humans in Europe are primarily the tick *I. ricinus* and, less frequently, *I. persulcatus.* Transmissibility to various hosts is influenced by various factors, tick-dependent (e.g., tick reproduction and population density and host preference) [12,13] and tick-independent (e.g., vertebrate reservoir abundance and extrinsic factors, such as climatic conditions, vegetation type and management, and host susceptibility, behavior, and abundance). Infestation of ticks by various bacterial pathogens induces changes in gene expression and impacts vector behavior, e.g., infestation with *Borrelia* spp. leads to longer questing and increased motility of the ticks [14]. Climate change is one of the main factors that may affect the spread of tick-transported pathogens and diseases, not only through directly influencing the life cycle and mating habits of these vectors but also through expansion of their populations and their introduction into new previously uninhabited regions [15,16].

The prevalence of Borreliosis is increasing in European surveillance reports. This, apparently, is mainly due to the expansion of tick populations, not only to northern areas of Europe, in which case climate change might be contributing [17], but also to countries in Southeastern Europe, attributable to tick or vertebrate reservoir population mobility and changes in distribution patterns [18]. A key factor in the vector–host transmission cycle is also the augmentation of the likelihood of exposure that is trending lately not only in rural but also urban areas, i.e., recreational and residential types of exposure, with recorded increases in vector populations in urban recreational areas and the organization of mass-participation events, ranging from music festivals to ultramarathons, which now rank as intriguing well-established features of recreation in the countryside [19,20,21]. Thasos is a busy touristic island of Northern Greece, in which, apart from the available camping facilities, a variety of outdoor activities, such as hiking and cycling tours, traditional cultural events, and music festivals, are readily organized. The island is busiest, and the frequency of hosting these events is highest, during the period in which the infection is presumed to have occurred. Moreover, Northern Greece, in general, and the prefecture of Kavala, where Thasos belongs, is a geographical area in which the ticks vectoring *Borrelia*, among other diseases, are known to thrive [22]. Moreover, in Greece, serological studies have reported low seroprevalence (up to 2.2%) for *Borrelia* in dogs but significant seropositivity in sheep, reaching 23.6%, as reported in a study from 318 tick-infested sheep [23]. Our patient was an outgoing, socially active college student studying in Northwestern Greece and returning to her home region earlier in the summer for her summer holidays.

A major caveat in the evaluation of the true prevalence of Borreliosis in European countries remains the absence of any identified public health surveillance system in a significant proportion of them (8/34, Greece being included among them) [24]. This leads to significant challenges in the introduction and implementation of wide-scale public health interventions. The importance of establishing such national surveillance systems lies in the ability to identify trends within a country, not really to report the actual burden of the disease within a specific region. Lyme disease is considered endemic in Albania, North Macedonia, Bulgaria, and Turkey. The reported incidence of *Borreliosis* in Bulgaria is about 6/100,000 of the population, whereas in Turkey, 75 cases of Lyme disease have been reported [25,26,27,28]. The principal tick vector for *Borrelia* species in Europe, *I. ricinus*, is prevalent in Greece and potently infesting wild resident birds (mostly small passerines). Interestingly, however, epidemiological studies have not detected *Borrelia* in the vectors, in contrast to ticks collected from countries neighboring to Greece, in which *Borrelia burgdorferi sensu lato* species complex was found to be prevalent. Indeed, *Borrelia afzelii* was found to be the predominant species in *I. ricinus* ticks collected from Bulgaria [29].

Key towards this goal of developing reliable epidemiological surveillance mechanisms is the establishment of a consensus regarding the *Borreliosis* case definition and reporting within different countries. For this purpose, the first European case definitions produced by the European Union Concerted Action on Lyme *Borreliosis* (EUCALB), an EU-funded initiative, were formulated after wide consultation across Europe by clinicians on the EUCALB Advisory Board [30]. According to them, a combination of both clinical features and laboratory findings is recommended in order to both assist treating physicians to make an accurate diagnosis and thus provide the appropriate treatment and for the reported cases to be used for meaningful epidemiological studies.

Diagnosis of Lyme disease is based on the presence of symptomatology, which may, however, be quite diverse and/or atypical, objective physical findings (such as the manifestation of the characteristic erythema migrans (EM) rash, facial palsy, or arthritis), and history of possible exposure to infected ticks in combination with laboratory work-up. The standard two-tier testing technique (an initial screening test, usually enzyme immunoassay—ELISA—followed by a Western blot for reactive, equivocal samples [31], and testing for immunoglobulin—IgM and/or IgG—of the *Borrelia burgdorferi sensu lato* complex) may be poor in terms of detecting early localized infection (<50%). However, it is highly sensitive (>99% sensitivity) in the case of late infection [32]. A modified two-tiered testing enzyme immunoassay algorithm is able to detect even more cases of early *Borrelia* infection in areas with high prevalence [33]. In general, a positive IgM and a negative IgG test result after the first month of infection usually indicate cross-reactivity. The titer of IgM antibodies usually decreases significantly 4–6 months after infection, while IgG antibodies can remain detectable for years [34]. However, IgM antibodies against *Borrelia* may persist for several years after the active infection [35] and may indicate incapacity of seroconversion due to lack of a robust T-dependent B cell response, which could also be attributed to the pathogen itself [36]. People with symptoms of early Lyme disease should be thoroughly examined for EM-reminiscent rashes and questioned about the potential manifestation of analogous skin lesions within the last 1–2 months. The presence of an EM rash and recent tick exposure (i.e., outdoor activities in a likely tick habitat with high prevalence of Lyme disease, within 30 days of the appearance of the rash) in some countries are sufficient to set the diagnosis. However, in the absence of explicit tick bite history, and/or atypical rashes, further confirmation of the diagnosis is warranted [37]. Although serology remains the mainstay of diagnosis, of seronegative Lyme disease has been reported. Moreover, persistent disease has been observed, despite prompt antibiotic treatment in patients who have become seronegative in response to treatment [38,39]. 

## 4. Conclusions

This is a case of symptomatic seroconversion against *Borrelia afzelii* in a region in which no cases of autochthonous Borreliosis have been described so far, despite the well-characterized prevalence of the disease in neighboring countries. Greece is currently still included among the European countries with an absence of organized Borreliosis surveillance reports. The validated presence of *Ixodes* spp. ticks, in association with the serological detection of antibodies against *Borrelia* in domestic animals, is clearly indicative that better surveillance systems are required to accurately detect and monitor the emergence of Lyme disease cases in the region, especially considering the rapid growth in the prevalence of Lyme disease and the risk of significant long-term health consequences of those infected.

## Data Availability

Data are contained within the article.
